# Bibliography

**Published:** 1895-03

**Authors:** 


### Bibliography.

---

Manual of Operative Technics. A Practical Treatise on the Elements of Operative Dentistry. By Thomas E. Weeks, D.D.S. Published by H. D. Justi & Son, Chicago, Ill., 1894.

The need of a more systematic course in dental procedures was long ago clearly demonstrated to the teachers in dental colleges, and there has been an intelligent effort in this direction in nearly all the higher class schools to attain this end.

The difficulties to be surmounted multiply in proportion to the size of classes, and the general inadequate provisions made for large numbers. The prevailing idea that the subjects and modes of treatment detailed in this book have had their origin in recent years is a mistake. This may have been true of some schools, but others have quietly originated plans leading directly to the same results as Dr. Weeks has so admirably described in this valuable book.

Dental college education has been a gradual development, but while slow the changes have been constant, so that it is not surprising that we find graduates of even ten years writing of things as they existed when they were students as though they still continued as part of the curriculum of the present.

The author of this volume evidently realizes this, for he says in his preface, "The desire to be helpful to both teacher and student has been the constant motive, and I hope . . . that the principles are so represented that all teachers may use them as a basis for their own particular methods."

While it is recognized that silhouettes are very valuable as a means of instruction, it seems to the writer that more stress is laid upon these than is warranted. The time spent in cutting down teeth and mounting them might be better employed, after one or two have thus been prepared, in some other and more instructive work. The charts made fully answer the purpose, as it is impossible to see the gain to the student unless it be in manual dexterity in the use of saw and file.

"Steel and instruments" are fully described in Chapter II., together with their uses.

The personal instruction given in Chapter III., on "Canal Treatment," is exceedingly valuable, and if it be not already adopted in colleges should at once be made part of the demonstrator's work. This applies with equal force to pulp-treatment, capping, etc., very clearly described in the chapters devoted to these subjects; but practical experience leads the reviewer to regard preliminary training, beyond practice in the use of tin in operative dentistry, unnecessary if not useless, and, with large classes, impracticable. From this point on the studies should be upon the living subject.

The manipulation of foils can be taught out of the mouth by the use of tin-foil, and we entirely disagree with the author in his estimate of this metal. He evidently does not comprehend its qualities, for he says, "For introduction into cavities strips of foil are rolled in a napkin, twisted into ropes or *folded* into ribbons, etc." Now, in the opinion of the writer, this is exactly *the way it should not be used.* Further, he says, "Its homogeneity depends upon wedging or interdigitation." This is a positive error, for tin-foil is capable of cohesion, and it is this property that makes this metal so valuable to the teacher in preliminary work. The management of cohesive as well as non-cohesive gold can be explained by it.

The remarks made in regard to silhouettes applies with equal force to drawing of teeth and modelling in clay. The student can acquire the forms of teeth just as well in practical experience. Our observation of the results of drawing and modelling by students, previously untrained in these branches, has been, as a rule, very far from creditable as a reproduction of form, the point aimed at in this work.

The aim of the book to develop a higher standard of preliminary training is beyond all praise, and while enthusiasm has naturally run to extremes in this technical work, it will have a positive influence for good upon all the colleges of the country, in that it will crystallize thought of many educators in this direction.

SAMMLUNG VON MIKROPHOTOGRAPHIEN ZUR VERANSCHAULICHUNG DER MIKOSCOPISCHEN STRUKTUR DER ZÄHNE DES MENSCHEN. Herausgegeben von Dr. dent. surg. A. Gysi und Dr. Med. C. Röse. Zürich, Schweiz. (Microphotographs of Dental Histology.)

There has certainly been nothing presented in the way of dental histology equal to these plates. As a piece of photographic art work they seem to be beyond criticism, and in the representation of detail in dental tissues nothing to be compared with them has been presented. Indeed, in a histological sense, the views of some portions of the minute structure of dentine is a surprise, especially in the clear presentation of the prolongations of the odontoblastic layer, Neumann sheaths, and Tomes's fibres.

The photographs are on enamelled bromide paper 7 x 7 inches square, and are pasted on thick card-board 11 x 16 inches. Each photograph is accompanied by a lithographed reproduction, with explanatory notes in German and English, so that teachers desiring these for class work will experience no difficulty.

The following is the list of subjects treated in this portfolio.

1. Upper Canine of Man with Pulp. Longitudinal ground section, prepared after Von Koch's petrifying method,—thirteen diameters.

2. Pulp from the Crown of a Human Superior Molar, showing the distribution of the nerves and blood-vessels; specimen colored with osmic acid. Longitudinal ground section after Von Koch's petrifying method,—thirty diameters.

3. Follicle of First Milk Incisor and the Germ of the Permanent Incisor, from human fœtus (thirty centimetres long); frontal section through the decalcified lower jaw; specimen colored with borax-carmine and bleu de Lyon,—fifty-five diameters.

4. Odontoblasts and Dentine, from the incisors of a new-born cat; specimen colored after Mährenthal's method (pyroligenous acid),—six hundred diameters.

5. Odontoblasts and Connective-Tissue Cells, from pulp of the crown of the first upper molar (child fourteen years); prepared from a decalcified longitudinal section through the crown, colored with hæmatoxylin,—six hundred diameters.

6. Odontoblasts, showing Tomes's Fibres entering the Dentinal Tubules: ground after Von Koch's petrifying method, specimen colored with acid fuchsin,—eight hundred diameters.

7. Dentinal Tubules and Interglobular Spaces, from a ground section through the crown of an incisor of man; specimen colored after Golgi's method (chromium silver),—five hundred and fifty diameters.

8. Gelatine-Yielding Fibrils of the Dentine, from a ground section through the root of a bicuspid of man; specimen colored after Golgi's method (chromium silver),—seven hundred diameters.

9. Dentinal Tubules from a First Molar (child of seven years). Transverse ground section, showing Neumann's sheaths of the dentine tubes and the enclosed Tomes's fibres; specimen colored after Golgi's method (chromium silver),—sixteen hundred diameters.

10. Transverse Ground Sections through Enamel (of man), four photographs of nine centimetres square, from an incisor and a molar,

—three hundred, six hundred, twelve hundred, and twelve hundred diameters.

11. Enamel of a Human Molar, showing the "stripes of Schreger," and the brown "parallel stripes of Retzius;" from a longitudinal ground section,—one hundred diameters.

12. Dentine, Normal (transparent) Cementum and Cementum of Hypertrophic Character; from the root of a human molar. Longitudinal ground section, treated with bichloride of mercury,—four hundred and twenty-five diameters.

The price of this portfolio is fixed until February 1 at six dollars, or thirty francs. The collection was received too late to enable our readers who may wish it to take advantage of this time limit, but by applying to Dr. Alfred Gysi, Zahnarzt, Börsenstrasse 14, Zürich, Switzerland, they may be able to secure it at a slight advance on this price.

Teachers in dental colleges should have this collection, and it would be exceedingly valuable in private offices as a means of instruction to patients.

This is the first portfolio, and is issued partly as an experiment. If sufficient encouragement be extended others will follow. The expense of such an undertaking is necessarily great.

A Compend of Dental Prosthesis and Metallurgy. By George W. Warren, D.D.S. With One Hundred and Twenty-nine Illustrations. P. Blakiston, Son & Co., Philadelphia, 1894.

Dr. Warren is becoming a prolific writer in certain fields of dental authorship, and has rapidly followed his "Compend of Dental Pathology and Dental Medicine" with "Richardson's Mechanical Dentistry," revised and rewritten, and now presents to dental readers, through the publishers, P. Blakiston, Son & Co., this compend of Dental Prosthesis.

It cannot justly be regarded as a compend, as that word is ordinarily applied, for it is rather a condensed statement of mechanical operations prepared in a form for ready reference.

The brevity required to accomplish this tends frequently, as in all compends, to obscurity. This is illustrated in the description of the investment of a piece in plaster and sand. "It is customary to incase the piece in the plaster mixture to the depth of from one-half to three-fourths of an inch, leaving only the lingual surface of the plate and teeth uncovered." As this is a very important part of the process a fuller explanation would have been better.

The same criticism may be applied to the "soldering process."

In the experience of the writer there is no part of mechanical dentistry that has given him more trouble in teaching than this, and it not infrequently occurs that students fail to acquire it. This may seem strange, as the philosophy of the operation is very simple, and should be readily comprehended. A full chapter on this would not be labor lost in future editions, for the process as explained in this would, it is feared, lead the inexperienced into difficulty.

We have not space to follow the author through mechanical dentistry proper, crown and bridge, continuous gum, and finally through an admirably condensed chapter on dental metallurgy, but it can safely be said of the book that through its two hundred and sixty-two pages there is apparently a continuous effort to make this a daily companion of the student,—a text-book always available for ready reference.

The author might in future editions enlarge on important matters to advantage and reduce on others; for instance, the space so generously given to "temperament" might be abbreviated very materially, as its value to students as a guide to the arrangement of teeth is very questionable.

Brook Farm. Historic and Personal Memoirs. By John Thomas Codman. Arena Publishing Company, Boston, Mass., 1894.

Very few of our readers, it is imagined, have not at some time been interested in the frequent allusions in general literature to "Brook Farm," and have desired an opportunity to make themselves familiar with its history. It is a gratification to know that one of our number, a Brook Farmer in his youth, has prepared this most readable volume to meet this growing demand.

The fact that some of the brightest of men and women of New England were residents and active workers on this farm is sufficient reason for noticing a book devoted to its history in a purely professional publication. The great difficulty with the professional or business man is that he becomes narrow by the contracting force of his special training, and needs the broader humanitarian outlook that such a publication gives to freshen his life.

It was fortunate for Dr. Codman that his early boyhood was spent at this place, as it enabled him to give to the world the only detailed and satisfactory account of the life on that now celebrated farm. The only regret felt on reading this interesting book is that the author has too little to say of the men and women who made it famous. This is probably due to the fact that he was very young when he became one of the Farmers, and then the majority of those enrolled as members at that time had their reputations to make.

Rev. George Ripley was the founder of Brook Farm, but it had its real origin in that upheaval of the moral and religious life so marked in the period from 1836 to 1846. Ripley became subsequently one of the principal editors of the New York *Tribune*, and the writer recalls with pleasure the eagerly sought-for reviews from his pen.

The men and women who took a more or less prominent part in this work, and became the leaders of thought at a later period, may be mentioned: Hawthorne, Thoreau, Emerson, Channing, Orestus A. Brownson, Margaret Fuller, George W. Curtis, Theodore Parker, and Charles A. Dana. The latter still survives, and is the active chief editor of the New York *Sun*. After Mr. Ripley's death Dana wrote of Brook Farm as follows: "The healthy mixture of manual and intellectual labor, the kindly and unaffected social relations, the absence of everything like assumption of servility, the amusements, the discussions, the friendships, the ideal and poetical atmosphere which gave a charm to life,—all these continue to create a picture towards which the mind turns back with pleasure."

We have not space to quote the good things Dr. Codman has gleaned from experience, notes, and memory, to make this very readable book, nor would that be necessary, dealing, as it does, with a phase of experience peculiar to the transcendental period of the middle life of the present century.

The author has no sympathy with the idea that "associations" are unfit for women, for he says, "It has been asserted that associations and communities may do well for men, but that women can never get along in them. The experience of Brook Farm testifies against the assertion. If ever there was a clear record of faithfulness and devotion, of sacrifice, of love of principle, and earnest, unselfish work for unselfish ends, the women toilers of Brook Farm can claim it and secure it without cavil."

It seems to the writer that the perusal of such a book is something more than a mere detail of daily, weekly, or yearly experiences in a community, but that it reaches out to an inspiration and incentive to a broader and more unselfish life than the world generally presents. The men and women who organized the Brook Farm movement have ordinarily been regarded as dreamers in their day and generation, and while this, in a degree, might be regarded as true, they, with self-sacrifice, attempted to demonstrate to an unbelieving world, in the language of John S. Dwight, that "the organization of attractive industry will be the reconciliation of spirit and matter, of religion and the world," and if this be true, Brook Farm and the communities that grew out of it were but practical pictures of the conditions of the human race for ages to come, for the future of civilization must approach this condition, if it is ever to outgrow the semibarbarism of the present.

A Compend of Materia Medica, Therapeutics, and Prescription-Writing, with Especial Reference to the Physiological Action of Drugs. By Samuel O. L. Potter, M.D., M.R.C.P.L. Sixth Edition, Rewritten and Enlarged. P. Blakiston, Son & Co., Philadelphia, 1894.

This compend has been fully reviewed in former editions, and we can only repeat our former good opinion of it as one of the best condensed statements of fact upon the subjects considered now in the market. Compends have their objections, and they are serious, inasmuch as they tend to superficial work, but for easy reference and refreshers of memory they have a value oftentimes superior to larger works.

The book has been enlarged by the addition of seventy-five additional articles, among these chloralamide, hypnal, hydrogen dioxide, phenacetin, phenocoll, etc., bringing it into conformity with the seventh revision of the United States Pharmacopœia (1890).

